# A Phenotypic Structure and Neural Correlates of Compulsive Behaviors in Adolescents

**DOI:** 10.1371/journal.pone.0080151

**Published:** 2013-11-14

**Authors:** Chantale Montigny, Natalie Castellanos-Ryan, Robert Whelan, Tobias Banaschewski, Gareth J. Barker, Christian Büchel, Jürgen Gallinat, Herta Flor, Karl Mann, Marie-Laure Paillère-Martinot, Frauke Nees, Mark Lathrop, Eva Loth, Tomas Paus, Zdenka Pausova, Marcella Rietschel, Gunter Schumann, Michael N. Smolka, Maren Struve, Trevor W. Robbins, Hugh Garavan, Patricia J. Conrod, and the IMAGEN Consortium

**Affiliations:** 1 Department of Psychiatry, Université de Montréal, CHU Ste Justine Hospital, Montreal, Canada; 2 MRC Social, Genetic and Developmental Psychiatry (SGDP) Centre, London, United Kingdom; 3 Central Institute of Mental Health, Mannheim, Germany; 4 Universitaetsklinikum Hamburg Eppendorf, Hamburg, Germany; 5 Institute of Psychiatry, King’s College London, United Kingdom; 6 Department of Psychiatry and Psychotherapy, Campus Charité Mitte, Charité, Universitätsmedizin Berlin, Germany; 7 Institute of Neuroscience, Trinity College Dublin, Dublin, Ireland; 8 Institut National de la Santé et de la Recherche Médicale, INSERM CEA Unit 1000 “Imaging & Psychiatry”, University Paris Sud, Orsay, France; 9 AP-HP Department of Adolescent Psychopathology and Medicine, Maison de Solenn, University Paris Descartes, Paris, France; 10 Centre National de Génotypage, Evry, France; 11 Rotman Research Institute, University of Toronto, Toronto, Canada; 12 School of Psychology, University of Nottingham, United Kingdom; 13 Montreal Neurological Institute, McGill University, Montreal, Canada; 14 Department of Psychiatry and Psychotherapy, Technische Universität Dresden, Germany; 15 Neuroimaging Center, Department of Psychology, Technische Universität Dresden, Germany; 16 Behavioural and Clinical Neurosciences Institute, Department of Experimental Psychology, University of Cambridge, United Kingdom; 17 Mannheim Medical Faculty, University of Heidelberg, Germany; 18 The Hospital for Sick Children, University of Toronto, Toronto, Canada; 19 Central Institute of Mental Health, Medical Faculty Mannheim / Heidelberg University, Department of Addictive Behaviour and Addiction Medicine, Manheim, Germany; 20 Departments of Psychiatry and Psychology, University of Vermont, Burlington, Vermont, United States of America; Bellvitge Biomedical Research Institute-IDIBELL, Spain

## Abstract

**Background:**

A compulsivity spectrum has been hypothesized to exist across Obsessive-Compulsive disorder (OCD), Eating Disorders (ED), substance abuse (SA) and binge-drinking (BD). The objective was to examine the validity of this compulsivity spectrum, and differentiate it from an externalizing behaviors dimension, but also to look at hypothesized personality and neural correlates.

**Method:**

A community-sample of adolescents (N=1938; mean age 14.5 years), and their parents were recruited via high-schools in 8 European study sites. Data on adolescents’ psychiatric symptoms, DSM diagnoses (DAWBA) and substance use behaviors (AUDIT and ESPAD) were collected through adolescent- and parent-reported questionnaires and interviews. The phenotypic structure of compulsive behaviors was then tested using structural equation modeling. The model was validated using personality variables (NEO-FFI and TCI), and Voxel-Based Morphometry (VBM) analysis.

**Results:**

Compulsivity symptoms best fit a higher-order two factor model, with ED and OCD loading onto a compulsivity factor, and BD and SA loading onto an externalizing factor, composed also of ADHD and conduct disorder symptoms. The compulsivity construct correlated with neuroticism (r=0.638; *p*≤0.001), conscientiousness (r=0.171; *p*≤0.001), and brain gray matter volume in left and right orbitofrontal cortex, right ventral striatum and right dorsolateral prefrontal cortex. The externalizing factor correlated with extraversion (r=0.201; *p*≤0.001), novelty-seeking (r=0.451; *p*≤0.001), and negatively with gray matter volume in the left inferior and middle frontal gyri.

**Conclusions:**

Results suggest that a compulsivity spectrum exists in an adolescent, preclinical sample and accounts for variance in both OCD and ED, but not substance-related behaviors, and can be differentiated from an externalizing spectrum.

## Introduction

There is a recent trend in psychiatry to identify neuroendophenotypes and move towards ‘dimensionality’ in order to better capture individual vulnerability to psychopathology and the high rate of comorbidity of psychiatric conditions [1,2]. For instance, compulsivity is a dimension which has attracted a growing interest in the recent years. It can be defined as a tendency to perform unpleasantlyrepetitive acts in a habitual or stereotyped manner [3,4]. Classically, the compulsive behavior is known to be repeated in order to prevent perceived negative consequences [3,4], but some have highlighted that the actions are also often carried out to alleviate or prevent anxiety and distress [2,5]. A broader, more inclusive definition would be that compulsivity entails actions inappropriate to the situation which persist, have no obvious relationship to the overall goal and often result in undesirable consequences; it reflects the aberrant dysregulation of stimulus-response habit learning [2]. It can be differentiated from obsessionnality, which describes the state of being preoccupied or occupied by a specific thought or act and represents the cognitive phenomenon related to compulsivity [6], which is observed at a behavioral level. 

Compulsivity represents behaviors common to numerous conditions, namely Obsessive-Compulsive Disorder (OCD) [4], but also Eating Disorders (ED) [7], with some clinical evidence showing it may account for the high rate of co-occurrence between these two disorders [8]. There is substantial evidence that OCD and ED do not co-occur by chance [7,9]. There is also evidence that OCD symptoms appear before ED and that OCD scores are not significantly higher in underweight versus normal-weight patients, suggesting that there is only a limited role for malnutrition in explaining obsessionality in ED patients [10]. 

Specific personality correlates have been found for compulsive symptoms. Obsessive-compulsive personality disorder (OCPD) tends to be comorbid with obsessive-compulsive disorder, Anorexia and Bulimia Nervosa [11-17]. Samuel and Widiger [18] reported a strong relationship between OCPD and two facets of the Five-Factor model of personality (NEO PI-R) [19], namely conscientiousness and neuroticism. Perfectionism, a subfacet of conscientiousness, was also found to be associated with both ED and OCD symptoms in college students [20]. 

The existence of a ‘compulsivity continuum’, with the prototypical disorder of compulsive behavior being OCD, has been hypothesised [2,4,5]. It comprises a group of disorders, also referred to as ‘OCD spectrum’ disorders, which includes Eating disorders [2,7,21-24]. Tourette’s syndrome is also part of this spectrum [2,21-23,25], as well as, to a variable extent, other ‘behavioral addictions’ (e.g. Gambling, Sex Addiction) [2,23], Body Dysphormic Disorder [21-25], Autistic Disorder [2,21,23,25] and Trichotillomania [21-23,25]. 

The compulsivity spectrum has also been hypothesized to include other disorders, particularly substance abuse (SA) [2,4] and binge drinking (BD) [26], both of which entail a compulsive pattern of use. Indeed, the progression from aberrant positive behavioral reinforcement (impulsivity) to negative reinforcement (compulsivity) in addiction is well documented in animal models [27]. A drug addiction cycle is described, in which a binge/intoxication phase, considered as an early impulsive phase, precedes a terminal withdrawal/negative affect phase, considered to be characterized by compulsivity [27]. The same switch from impulsivity to compulsivity has been found in rats prone to compulsive cocaine self-administration [2,28]. Furthermore, in animal models of stimulant drug abuse, individual differences in impulsivity are understood as a predisposing factor to compulsive drug taking [2]. It is worth noting that high impulsivity was not shown to play a role in loss of control of heroin intake in rats, as it had been for cocaine [29]. Nevertheless, both obsessionality and compulsivity have been described in opioid addicted individuals [30]. Hence, Robbins et al. [2], while proposing two transdiagnostic dimensions, impulsivity and compulsivity, as endophenotypes to explain comorbidity between psychiatric disorders, hypothesized that both impulsivity and compulsivity were involved in substance use disorders [2]. Even though substance use offers short term pleasure that is not seen in OCD, substance use disorders and OCD share compulsive behaviors that are reinforced by relieving distress [2,23]. 

As mentioned, while SA and BD have been implicated in this compulsivity continuum and have been shown to involve a compulsive aspect, there is stronger evidence for their involvement in an externalizing dimension, which also includes conduct problems and ADHD. Indeed, the high rate of comorbidity between substance-use disorders, conduct disorder (CD) and attention-deficit/hyperactive disorder (ADHD) is well-documented [31]. A number of studies support the validity of a general externalizing spectrum model which accounts for the common variance between antisocial behavior, CD, ADHD, SA and BD, and can be explained by common genetic and environmental influences, and pre-morbid personality traits, namely impulsivity and novelty seeking [32-34].

There is indirect evidence that these behavioral spectra also differentiate at a cognitive and neural level. Prefrontal cortex sub regions, such as the orbitofrontal cortex (OFC), have been consistently linked to OCD [5,35-37]. The OFC has been found to have a role in cognitive flexibility and, particularly, reversal learning, which is the ability to adapt a behavior after negative feedback [4]. A deficit in reversal learning, for example, created by a lesion of the OFC, is believed to contribute directly to compulsivity [4]. Both positron emission tomography (PET) and fMRI studies show altered OFC activity in OCD patients [35]. There is also robust evidence for changes of OFC volume in these subjects in structural MRI studies [38-44]. Many Voxel-Based Morphometry (VBM) studies also pointed to OFC volume changes in OCD [35,45,46], which was confirmed by a more recent meta-analysis of ten VBM studies demonstrating gray matter volume changes in the OFC-striatal loop [37]. Many gray matter volume studies [47,48] and functional neuroimaging studies [49-52] have also directly implicated the OFC in ED.

Some reviews have hypothesized a direct link between OFC dysfunction and the compulsivity dimension [2,4,5]. Similarly, the right inferior frontal cortex (IFC) and right inferior frontal gyrus (IFG) have been implicated in impulsivity [2,53,54]. The role of the right IFC in response inhibition (using Stop-Signal or Go/No-Go tasks) is well documented in the literature [55-63], implicating the involvement of the right IFC directly in impulsivity, which can be viewed as pre-potent motor disinhibition [4]. Many fMRI and EEG studies have also highlighted the role of the right IFC in disinhibited responding characteristic of individuals with ADHD [64-70] and substance use profiles [2,71-75].

While the neural correlates of impulsivity and the externalizing spectrum and its relationship to the right IFG have already been investigated [76], no study has yet investigated the neurocognitive correlates of the variance common across disorders of the compulsivity spectrum. Further, Maia, Cooney et al. [77] point to the importance of studying presymptomatic cases when investigating neurocognitive mechanisms of OCD and related disorders due to the possibility that differences between patients and controls could also reflect consequences of the long-standing illness, and its treatment.

### The Current Study

The current study aimed to use multivariate statistical modelling to first examine the validity of the hypothesized compulsive spectrum across OCD, ED, SA and BD symptoms in a subclinical adolescent population. As described earlier, substance abuse and binge drinking have already been validated as being part of an externalizing behaviors phenotypic structure. Taking this into account, we propose to model symptoms associated with compulsivity while accounting for an externalizing spectrum (ADHD, SA and CD) to test whether a compulsive spectrum can be clearly differentiated from a more impulsive externalizing spectrum and to investigate how variance is shared between them. 

In line with this, the next step was to examine whether each latent construct was associated with distinct personality dimensions. We hypothesized that NEO-FFI conscientiousness and neuroticism would predict the compulsivity construct, and that extraversion and TCI novelty-seeking the externalizing behaviors construct. Finally, the neural correlates of these two dimensions using voxel-based morphometry (VBM) analysis of brain gray matter volume were examined. We hypothesized that, in a large community sample of adolescents, the compulsivity construct would be associated with alterations in OFC volumes, whereas the externalizing behaviors construct would be associated with altered IFG volumes. 

## Methods and Materials

### Ethics Statement

The study was conducted in accordance with the Declaration of Helsinki and the IMAGEN protocol was centrally approved by the King’s College London Research Ethics Committee and each local institutional ethics committee (Central Institute of Mental Health, Mannheim; Charité Universitätsmedizin, Berlin; University Medical Center,Hamburg-Eppendorf; University of Nottingham, Nottingham; Trinity College, Dublin; Institut National de la Santé et de la Recherche Médicale, Orsay). A multidisciplinary ethics committee within the consortium also provided guidance on procedures (for example, consent, confidentiality and data protection) involving vulnerable groups (adolescents) and strategies to deal with sensitive issues related to novel findings of the contribution of genetic, biological and environmental factors in personality and psychopathology in an ethical manner. Parents gave informed written consent and adolescent provided written assent to the study procedure before their participation.

### Participants and Procedure

A community-based sample of young adolescents (N=2000) was recruited for the IMAGEN study (for details on the IMAGEN project, see Schumann et al. (2010) [78]). In brief, participants and their parents were recruited via high-schools in 8 European study sites. The geographical areas were selected to have minimal ethnic diversity to maximize homogeneity as a prerequisite for future genome-wide association analyses. Private and state-funded schools were targeted in order to obtain a diverse sample of socio-economic status, emotional and cognitive development. The IMAGEN study included, in the first phase, a home assessment using an online computer platform (described below) and 1 or 2 study-centre visit.

After data quality control, complete and reliable data sets of personality, psychiatric symptoms and substance use behaviors were available for 1938 participants with an average age of 14.5 years (SD=0.4) and an even gender ratio (n=984 girls, i.e. 50.8%). According to the research site in England (London and Notthingham), Ireland (Dublin), Germany (Berlin, Hamburg, Mannheim and Dresden) or France [79], the tests were administered in English (37.8%), in French (12.4%) or in German (49.9%). Of these volunteers, 1639 had complete neuroimaging data. 

### Measures

Brevity, age-appropriateness and validity in the three languages (English, German and French) were the basis of selection for all measures.

### Psychiatric symptoms

Psychiatric symptoms were assessed using the validated Development and Well-Being Assessment (DAWBA) interview [80], which was administered at the research site. The diagnostic criteria were based on the DSM-IV [81]. OCD and ED were assessed by using the DAWBA interview- child report, whereas for ADHD and CD parent’s reports were preferred, and were thought to be more reliable. For each disorder, a composite score was created by adding all the questions relating to symptoms (e.g., over the last 4 weeks have you engaged in any of the following rituals?: excessive cleaning (hand washing, baths, showers, toothbrushing, etc.); scored as No (0), A little (1) or A lot [82]). A second composite score was created for each disorder using impact questions (e.g. Have the rituals or obsessions interfered with how well you get on with the rest of the family?; values ranged from Not at all (0) to A great deal [82]). Exceptionally for ED, an additional score was used representing the sum of two general, screening questions (e.g. Have you ever thought you were fat even when other people told you that you were very thin?). Bands scores, representing the likelihood of having a disorder, were also used as measured variables for each disorder. For ED, the band score represented the likelihood of having a general eating disorder, and was not specific to a subtype.

### Substance Use behaviors

Substance and alcohol use were assessed via the online computer Psytools ® (Delosis Ltd, London, UK) platforms at the participant’s home. Binge drinking was assessed using the Alcohol Use Disorders Identification Test (AUDIT) [83]. AUDIT frequency and AUDIT problematic use were used as drinking measures and a binge drinking composite score was created by summing 13 items from the European School Survey Project on Alcohol and Drugs (ESPAD) [84,85] on alcohol quantity (e.g. how many drinks containing alcohol do you have on a typical day when you are drinking"), frequency of drinking (e.g. On how many occasions in your whole lifetime have you had any alcoholic beverage to drink?), binge drinking (e.g. How many times in your whole lifetime have you had five or more drinks in a row?) and intoxication episodes (e.g. How many drinks do you usually need to get drunk), as well as items on expectation for the next binge drinking episode (e.g. How likely is it, if you drink alcohol, that you would not be able to stop drinking?).

Two illicit substance use scores were derived from the ESPAD [84,85]. The first, age of onset of consumption for any drug (marijuana or hashish, inhalants, tranquilizer or sedatives, amphetamines, LSD, magic mushrooms or hallucinogens, crack, cocaine, heroin, narcotics, ecstasy, ketamine or phenylclinidine, GHB or liquid ecstasy and anabolic steroids), was coded inversely (’16 years old=1’ down to ’11 years old or less=6’) to indicate positive associations between higher levels of risky behavior and earlier onset of use. The second score was a composite of frequency of cannabis use (according to seven response options ranging from ‘0’ to ’40 or more’ times) in a lifetime, past year, past month and the past week. 

### Personality traits

Both personality questionnaires used in this study were administered at home with the Psytools ® platform. The NEO-Five Factor Inventory (NEO-FFI) [19] was used to assess three characteristics of interest: neuroticism, conscientiousness and extraversion. The Temperament and Character Inventory (TCI-R) [86] was used to assess novelty seeking, which is considered a good measure of impulsive tendencies [63,87].

### MRI data

An overview of the specifications, quality control and standardization across sites can be found elsewhere [78]. For analysis of structural neuroimaging data, gray matter volumes from 1639 adolescents were utilized. 

### Data Analysis

To examine the relationship between OCD, ED, BD and SA symptoms, two structural equation models were tested. The first was a one-factor model, with all symptoms co-loading onto the same general compulsivity/externalizing behaviors factor, to assess how the variance was shared between the disorders. The second model was a higher-order two-factor model, derived from the results of the first model. All analyses were carried out using Mplus version 5.21 [88] and Maximum Likelihood with Robust standard errors (MLR) estimation. Unlike Maximum Likelihood , MLR does not depend on assumptions of normality and thus has been shown to perform well when modelling low prevalent behaviors or non-normal data [89]. Test of goodness of fit included the Comparative Fit Index (CFI), the Root Mean Square Error of Approximation (RMSEA) and the Standardized Root Mean square Residual (SRMR). Thresholds used to assess goodness of fit are traditionally CFI>0.90, RMSEA<=0.05 and SRMR<0.05. Models were compared using Akaike’s information criterion (AIC) and the Bayesian information criterion (BIC), both useful in comparing two non-nested models. The best balance of fit and parsimony is illustrated by smaller values in both of these indices. Path loadings were considered significant for a value above 0.3. The best fitting model was then controlled for gender, language, and site. The language variable was orthogonally transformed into two variables, which were both entered into models, as was Gender (with female=0 and male=1), while site was entered as a cluster variable. Personality correlates were then added to the best fitting structural model. 

The gray matter image pre-processing followed the optimized VBM protocol implemented in Statistical Parametric Mapping (SPM) [90]. Pre-processing incorporated image registration and classification into a single generative model [91]. Segmented gray matter data were scaled by the inverse-Jacobian of the local transformations (i.e., modulated) in order to preserve volume. The spatially normalized (standard MNI space) and modulated gray matter partitions were smoothed using a 8 mm full-width at half maximum Gaussian kernel. Only voxels with an absolute value greater than 0.05 were included in the statistical analysis. 

The factor scores were extracted (using SPSS 19 software) in order to look at neural correlates using VBM analysis. Total gray matter volume, age, sex, handedness, pubertal development status and site were entered as nuisance covariates. Externalizing behaviors factor scores were included as nuisance variables when investigating compulsivity factor scores, and vice versa. Both positive and negative correlations with each factor score were examined. A voxel-wise threshold correcting for multiple comparisons and controlling for family-wise error (FWE) rate was calculated using 3dClustSim; a Monte Carlo simulation implemented in AFNI [92]. This deterministic sampling algorithm ascertains the frequency of significant clusters that would occur by chance under the null hypothesis (i.e., the false positive rate) based on its size, the level of smoothness associated with the data, and 10,000 random image permutations. Significant voxels passed a voxelwise statistical threshold (*t*(1629) = 2.58, *p* < 0.005) and were required to be part of a larger 350 mm^3^ cluster of contiguous significant voxels, giving a 0.05% probability of a cluster surviving due to chance.  

See Table A and Table B in supplementary material (File S1) for zero-order correlations between all variables included in analyses. The distribution (frequencies of having any symptoms, mean, standard deviation and skewness) of key variables (symptom composite scores for each disorders, binge drinking and substance abuse, as well as personality correlates) were also added as annex (Table C in File S1). 

### Missing Data

Missing data represented between 0 to a maximum of 0.8% in measured variables for psychiatric symptoms, and between 3 to 9.7% for binge drinking and substance use. Full information likelihood (FML) was used to account for missing data in analysis. 

## Results

Two higher-order structural models were tested to examine the nature of the relationship between OCD, ED, BD and SA, and whether they, indeed, belonged to a compulsive spectrum. CD and ADHD symptoms were used in the model to represent the externalizing behaviors spectrum. First-order latent variables were modeled for each type of psychiatric disorders: OCD (symptoms (OCD1), impact (OCD2) and band (OCD3)), ED (screening (ED1), symptoms (ED2), impact (ED3) and band (ED4)), BD (frequency (BD1), problem (BD2) and ESPAD composite (BD3)), SA (age of first try composite score (SA1) and cannabis composite score (SA2)), ADHD (symptoms (ADHD1), impact (ADHD2) and band (ADHD3)) and CD (symptoms (CD1), impact (CD2) and band (CD3)). For example, OCD1, OCD2 and OCD3 served as indicators for the latent variable OCD. This base model, with independent factors representing each set of symptoms, served as the bases for the two higher-order models. This base model did not fit the data well (fit statistics for all models tested are reported in Table 1), suggesting that significant common variance is shared by all or some of these factors.

**Table 1:** Data fit for the three models (N=1938).

**Models**	**X^2^**	**Df**	**CFI**	**AIC**	**BIC**	**RMSEA**	**SRMR**
**Separate Sub-factors Model**	1914.89	125	0.82	117098.59	117455.03	0.09	0.11
**Higher-Order One Factor Model**	786.17	129	0.93	115126.31	115460.48	0.05	0.08
**Higher-Order Two Factor Model**	726.24	128	0.94	115034.65	115374.39	0.04	0.06

**Figure 1:**
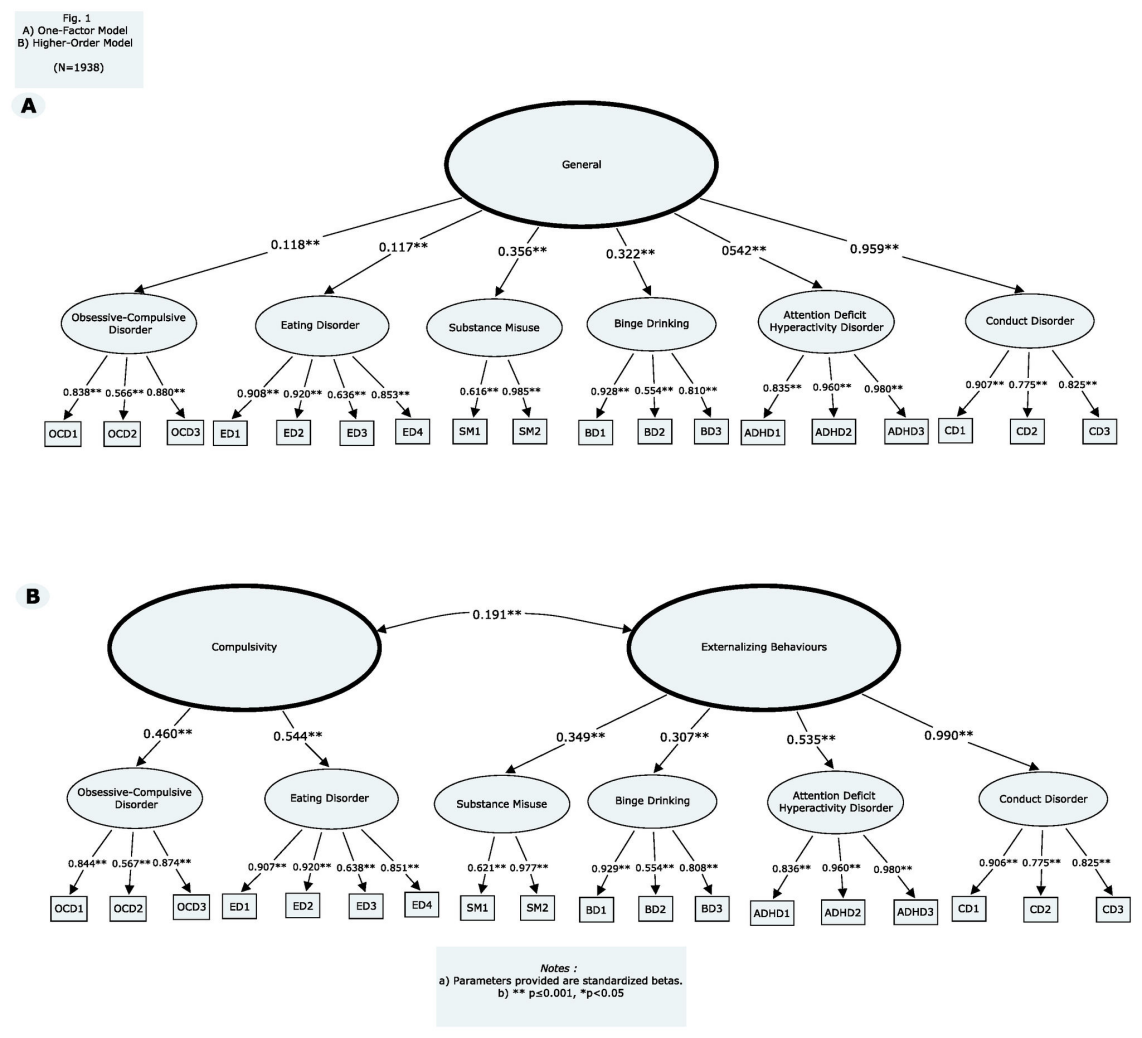
One-factor model (A). Higher-order model (B). (N = 1938).

A higher order one factor model was first tested and was composed of a second-order general construct with loadings on all six first-order latent variables (OCD, ED, BD, SA, CD and ADHD). The higher-order one factor model, along with path loadings, is represented in Figure 1a. The model fit the data acceptably (see table 1). Although all loadings were significant (p<0.001), two paths were below the generally accepted cutoff of 0.3, namely OCD (r=0.109) and ED (r=0.092). This result suggested that OCD and ED would load onto a different latent factor, whereas BD, SA, CD and ADHD share common variance and would load onto the same construct.

A second, higher-order two factor model was then tested, with ED and OCD loading onto a compulsivity (COMP) construct, and BD, SA, CD and ADHD loading onto an externalizing behaviors (EB) construct. This model and its path loadings are schematically represented in Figure 1b. This second higher-order model fit the data well and, in comparing all fit criteria, was the better fitting model. Compulsivity and externalizing behaviors construct were allowed to correlate (r=0.191; *p*≤0.001). In this model, residuals for ADHD and CD, as well as SA and BD were allowed to correlate, as was suggested by the modification indices (131.87 and 150.72, respectively), and considered justifiable as they were assessed with items from the same instrument. This improved model fit (X^2^=546.76, df=126, CFI=0.96, AIC=114764.22, BIC=115115.09, RMSEA=0.04, SRMR=0.04). Gender, site and language were controlled for next. This final model fitted the data well (X^2^= 853.61, df=172, CFI=0.95, AIC=126746.89, BIC=127192.44, RMSEA=0.05, SRMR=0.04). All path loadings remained significant and ranged from 0.457 to 0.660 (*p*≤0.001), except for ADHD with a loading of 0.231 (*p*=0.000).

### Personality Correlates

**Figure 2:**
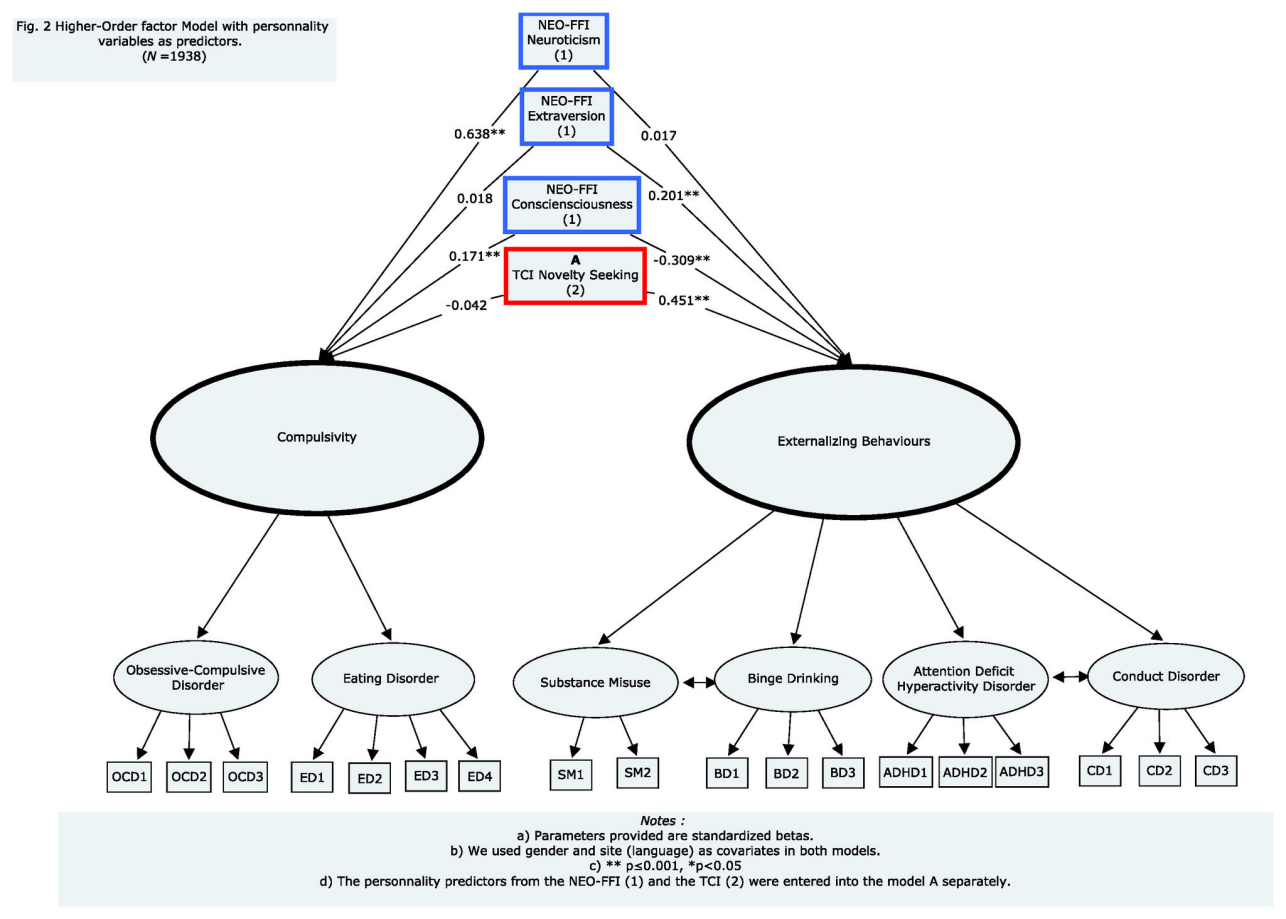
Higher-order model with personality correlates as predictors. (N = 1938).

Regression coefficients (Figure 2) showed that neuroticism was associated positively with COMP (r=0.638; *p*≤0.001), but not with EB (r=0.017; *p*=0.737). Conversely, extraversion was associated positively with EB (0.201; *p*≤0.001), but not with COMP (r=0.018; *p*=0.680), whereas conscientiousness was associated positively with COMP (r=0.171; *p*≤0.001), and negatively with EB (r=-0.309; *p*≤0.001). Finally, novelty seeking was associated positively with EB (r=0.451; *p*≤0.001), but not with COMP(r=-0.042; *p*=0.494). 

### Neural correlates

**Figure 3:**
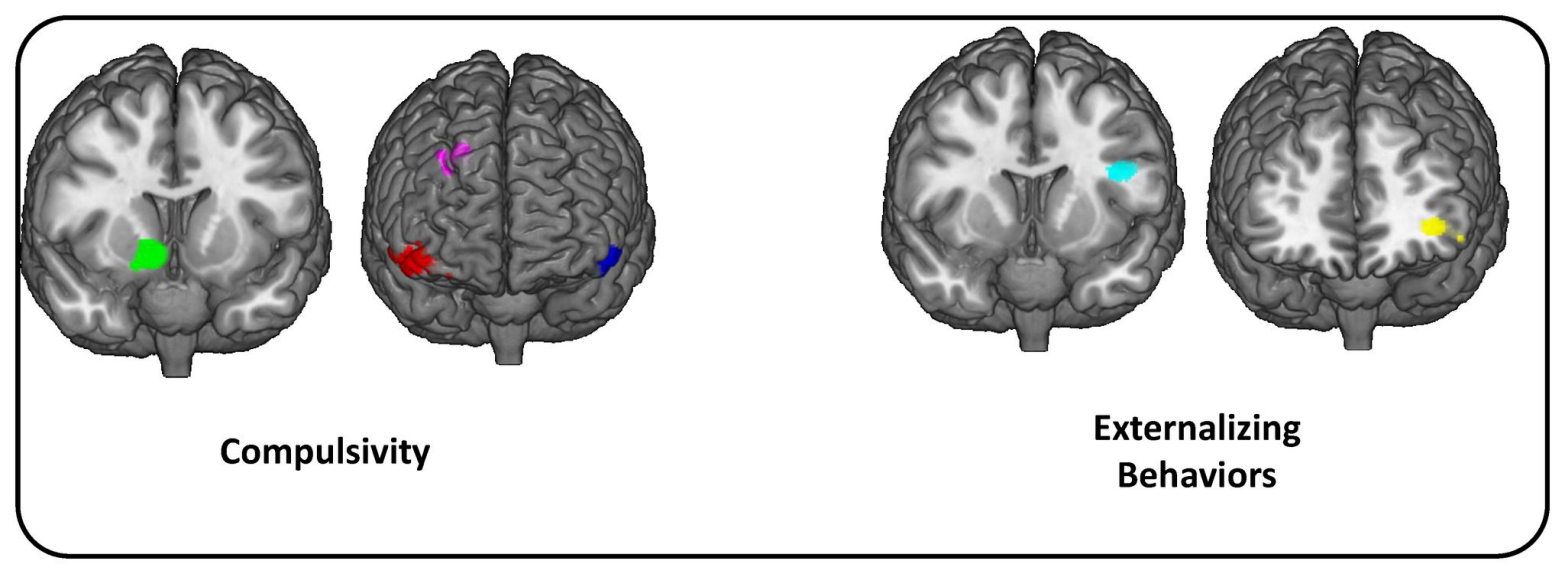
Anatomical locations for the four significant clusters correlated with Compulsivity and three correlated with Externalizing Behaviors identified in the VBM analysis.

 The coordinates, anatomical locations, and peak t value for the VBM analysis are given in Table 2. The VBM analysis identified four significant clusters that were positively correlated with COMP, namely the left and right orbitofrontal cortex, the right ventral striatum (VS) and right the dorsolateral prefrontal cortex (DLPFC). Three clusters were significantly negatively correlated with EB: the left inferior frontal cortex in Broadman’s area 9 and 10, as well as the middle frontal gyrus (Figure 3). 

**Table 2:** Coordinates, anatomical locations, and peak t value for the VBM analysis of compulsivity and impulsivity factors (N=1639).

**Latent Factor**	**Correlation Positive (P) Negative (N)**	**Right(R)Left(L)**	**Anatomical region**	**Peak voxel coordinates (MNI)**			**Peak t**
				x	y	z	
**Compulsivity**	P	R	OFC	54	33	-18	5.46
**Compulsivity**	P	R	Ventral striatum	12	6	-6	4.38
**Compulsivity**	P	L	OFC	-54	34.5	-16.5	4.84
**Compulsivity**	P	R	DLPFC	21	43	42	4.09
**Externalizing**	N	L	IFG (BA10)	-39	46.5	-1.5	4.42
**Externalizing**	N	L	IFG (BA9)	-43.5	10.50	22.5	4.10
**Externalizing**	N	L	MFG	-22.5	34.5	39	3.42

*OFC: orbitofrontal cortext; DLPFC: dorsolateral prefrontal cortex; IFG: inferior frontal gyrus*;

*BA: Brodmann’s area; MFG: middle frontal gyrus.*

## Discussion

The first aim of this study was to test the validity of a compulsivity spectrum and to determine which disorders and psychiatric symptoms it would include, and if it would distinguish itself from an externalising spectrum in a community sample composed of young adolescents. Results showed that eating and obsessive-compulsive disorders symptoms explained a compulsivity construct, whereas substance abuse and binge drinking manifestations belonged to an externalizing behaviors construct, also formed of conduct disorder, and to a lesser extent, attention-deficit/hyperactive disorder symptoms. These findings in support of a compulsivity construct are consistent with the hypothesized compulsivity continuum proposed byFineberg et al. [4],Robbins et al. [2] and Grant et al. [5]. The second part of this model, which represented ADHD, CD, SA and BD loading onto an externalizing behaviors construct, resembled, although it was not identical, to models already found in the literature [32,33], where substance abuse and binge drinking were found to be part of an externalizing continuum. These findings support the theory that compulsivity is less implicated in drug abuse initiation, and more at a later stage of addiction, when preventing negative affect/withdrawal symptoms becomes an important motive for drug-taking [27,29], which may not be the case in this young subclinical sample. Hence, while other studies suggest that compulsivity might be implicated in later stages of drug addiction and specific addiction profiles (e.g., cocaine dependence)[27,29], our findings only support a correlation with an impulsivity/externalizing dimension in this adolescent sample . 

The second aim of this study was to examine the personality correlates of the compulsivity continuum. Results showed that neuroticism and conscientiousness positively predicted the compulsivity construct. Both neuroticism and conscientiousness have been consistently linked with OCPD [18] which, in turn, has been hypothesized to be part of this compulsivity continuum [4,21]. Moreover, ‘disinhibited’ personality characteristics, such as extraversion and novelty seeking predicted externalizing behaviors, but not the compulsivity latent factor, suggesting distinct personality endophenotypes for each spectrum. Similar results were reported by Castellanos et al. [33], who found impulsive personality traits longitudinally predicted an externalizing behaviors construct. Additionally, conscientiousness correlated negatively with the externalizing behavior construct, further differentiating the two dimensions. These results provide discriminant validity for the two dimensions modelled in this study and suggest personality traits as potentially underlying the common variance between highly co-morbid disorders. 

The third aim of the study was to identify neural correlates of these psychiatric endophenotypes, and this study provides strong support for the hypothesized orbitofrontal abnormalities in compulsivity [2,4,5] and also implicated the OFC in eating disorders [48,50-52]. More particularly, this study echoes the robust evidence for the role of OFC in OCD [5,35,36], described in both structural MRI [38-42] and VBM studies [37,45,46], with older, clinical populations. The increased gray matter of the OFC in this study of a young sample could be construed to be associated with a preclinical phase of compulsivity, before treatment and evolution of the disease. Coincidentally, the increased gray matter volume in the ventral striatum associated with compulsivity is not surprising, given that striatal gray matter volume changes have been associated with both ED [47] and OCD [35,46] in the literature. These results are consistent with the theoretical ‘orbitofronto-striatal model’ behind both OCD [35] and the compulsivity dimension. 

The positive relationship found between compulsivity and gray matter volume in the dorsolateral prefrontal cortex was not anticipated, but has resonance in the compulsive disorders literature. The DLPFC has a well-documented involvement in cognitive flexibility [93,94]. DLPFC changes have also been widely associated with OCD [35,36] and have been described in ED [48,95,96] as well. This study has echoed results found by Sakai et al. [97] who found functional connectivity with the ventral striatum to be significantly increased in the OFC, the DLPFC, and the ventromedial prefrontal cortex in non-medicated OCD patients compared to controls. The implication of these regions can now be extended to a compulsivity continuum that can be measured in an adolescent population. The results found in this study point specifically to functioning in the OFC, DLPFC and VS as neurocognitive endophenotypes of compulsive behaviors.

Consistent with previous literature, frontal gyrus gray matter volume was negatively correlated to the externalizing behaviors construct, but this contradicts some studies that have highlighted the involvement of the right IFC more specifically in impulsivity [2,4,87] and in disorders of the externalizing spectrum such as ADHD [64-66]. However, there exists increasing evidence in the literature of bilateral involvement of the IFG in response inhibition [56,73,98], in ADHD [64,99,100] and in substance abuse [71,72]. Furthermore, Swick et al. [101] showed the left IFG to be critical for successful inhibition on a Go/NoGo trial in patients with cerebral lesions in this region, despite the focus on right IFG in neuroimaging studies. The negative association found between left middle frontal gyrus gray matter volume and the externalizing behavior construct, even if not part of the initial hypothesis, is consistent with the impulsivity literature, with MFG correlating with response inhibition [57,71], particularly in adolescent and adult subjects with substance abuse profiles [72,73]. 

Impulsiveness in adults has also been shown to be negatively correlated with cortical thickness in the MFG [102]. Moreover, a recent VBM study found an association between trait impulsivity and gray matter volume in both the left IFG and MFG of cocaine dependent individuals, a pattern directly opposed to the association described in controls [103]. Similarly to compulsivity, impulsivity has been linked in the literature to a cortico-striatal loop, as well as diminished striatal activation to rewards [2,4,5]. Although this study replicated well-known associations between impulsivity and cortical correlates, it did not identify any striatal involvement. Striatal abnormalities may have been better detected by functional imaging coupled with a specific task involving reward-related behavior [4]. The absence of striatal implication could also be linked to the fact that the current data seems to represent a preclinical phase of externalizing behaviors disorders. Nevertheless, this study showed a clear differentiation between the compulsive and externalizing spectrums.

One strength of the study is its large sample, including an equal proportion of both male and female adolescents from four different countries, which allows the results to be applicable to a wide range of adolescent populations. This study examined transdiagnostic behavioural and neural markers, which, according to many, will allow for new genetic and therapeutic approaches in psychiatry [2]. Furthermore, one of the major interests of the current study was to do so in a subclinical sample, so that results would not be influenced by a long-standing illness or its treatment. Identifying neuroendophenotypes in presymptomatic cases can eventually lead to early detection of the disorders before its complete expression [2], and to the development of preventive tools. Researching neuroendophenotypes could also provide a more quantitave measure of the deficits, relying less on clinical ratings alone, and improving the use of informative animal models while studying neural processes present across species [2]. The variety of the available data for this sample, including genetic material, is another strength of this study. Genetic correlates could be added in the model as a future aim. 

### Limitations

One limitation of this study is the low prevalence of psychiatric disorders in this normal sample which prevented us from testing the role of other disorders of the compulsivity spectrum such as Tourette’s Syndrome. The tools used in this study, as well as the age of the subjects, also prevented the inclusion of disorders such as Gambling and Body Dysmorphic Disorder. The ethnic homogeneity, useful for genetic studies, could have restricted applicability of the results. Another limitation is that the tool used to assess psychiatric symptoms, the DAWBA, did not allow the identification of eating disorders subtypes. Some have hypothesized impulsive tendencies, as well as compulsivity in Bulimia Nervosa more specifically [2,104-106]. However, the aim of this study was to look at subclinical symptoms and to bring to light a compulsivity dimension, rather than to study specific diagnosed disorders. Also, it has been hypothesized that the link between compulsivity and addiction might manifest at advanced stages of substance dependence [27], and that there may be different predispositions and trajectory of addiction depending on type of drug used [27,29]. Our findings suggest that at early stages of substance use and misuse, compulsivity does not account for alcohol and drug-related behaviors. The low prevalence of certain types of drug use in this young sample did not allow for an examination of specific associations with different drugs. It would be worthwhile to further test this model in an adult clinical sample where substance use and mental health symptoms are more severe and hypotheses about the relationships between compulsivity and the later stages of these disorders can be tested.

## Supporting Information

File S1 Table A. Correlations and descriptive statistics for study variables (N=1938). Table B. Correlations between covariates (personality and neural) with compulsive an externalizing behaviors (N=1938). Table C. Frequencies and distribution of compulsive and externalizing symptoms, as well as personality correlates (N=1938).
(DOC)
