# Supplementary material for: A Phenotypic Structure and Neural Correlates of Compulsive Behaviors in Adolescents
Source: PLoS One. 2013 Nov 14;8(11):e80151. doi: 10.1371/journal.pone.0080151 (PMC3828212; doi:10.1371/journal.pone.0080151)
Supplement: File S1 — Table A. Correlations and descriptive statistics for study variables (N=1938). Table B. Correlations between covariates (personality and neural) with compulsive an externalizing behaviors (N=1938). Table C. Frequencies and distribution of compulsive and externalizing symptoms, as well as personality correlates (N=1938). (DOC) [file pone.0080151.s001.doc]

Table A. Correlations and descriptive statistics for study variables (N=1938).

|  | 1 | 2 | 3 | 4 | 5 | 6 | 7 | 8 | 9 | 10 | 11 | 12 | 13 | 14 | 15 |  | 16 | 17 | | 18 | |
| --- | --- | --- | --- | --- | --- | --- | --- | --- | --- | --- | --- | --- | --- | --- | --- | --- | --- | --- | --- | --- | --- |
| 1. OCD symptoms |  |  |  |  |  |  |  |  |  |  |  |  |  |  |  |  |  | |  | |  |
| 2. OCD impact | 0.48** |  |  |  |  |  |  |  |  |  |  |  |  |  |  |  |  | |  | |  |
| 3. OCD band | 0.74** | 0.50** |  |  |  |  |  |  |  |  |  |  |  |  |  |  |  | |  | |  |
| 4. ED general | 0.20** | 0.09** | 0.19** |  |  |  |  |  |  |  |  |  |  |  |  |  |  | |  | |  |
| 5. ED symptoms | 0.20** | 0.13** | 0.18** | 0.83** |  |  |  |  |  |  |  |  |  |  |  |  |  | |  | |  |
| 6. ED impact | 0.20** | 0.23** | 0.16* | 0.55** | 0.62** |  |  |  |  |  |  |  |  |  |  |  |  | |  | |  |
| 7. ED band | 0.19** | 0.09** | 0.19** | 0.79** | 0.78** | 0.52** |  |  |  |  |  |  |  |  |  |  |  | |  | |  |
| 8. CD symptoms | 0.05* | 0.04 | 0.07** | 0.07** | 0.27* | 0.09** | 0.11** |  |  |  |  |  |  |  |  |  |  | |  | |  |
| 9. CD impact | 0.05* | 0.02 | 0.08** | 0.04 | 0.06** | 0.06* | 0.07** | 0.71** |  |  |  |  |  |  |  |  |  | |  | |  |
| 10. CD band | 0.08** | 0.08** | 0.11** | 0.08** | 0.07** | 0.09** | 0.11** | 0.75** | 0.61** |  |  |  |  |  |  |  |  | |  | |  |
| 11. ADHD symptoms | 0.04 | 0.04 | 0.11** | 0.00 | -0.04 | 0.03 | 0.02 | 0.47** | 0.50** | 0.41** |  |  |  |  |  |  |  | |  | |  |
| 12. ADHD impact | 0.05* | 0.01 | 0.08** | -0.01 | -0.04 | 0.02 | 0.03 | 0.45** | 0.58** | 0.39** | 0.79** |  |  |  |  |  |  | |  | |  |
| 13. ADHD band | 0.05* | 0.04 | 0.12** | 0.00 | -0.03 | 0.03 | 0.03 | 0.44** | 0.48** | 0.039** | 0.94** | 0.82** |  |  |  |  |  | |  | |  |
| 14. Audit frequency | 0.04 | 0.02 | 0.04 | 0.10** | 0.12** | 0.03 | 0.10** | 0.23** | 0.12** | 0.30** | 0.05* | 0.05* | 0.05* |  |  |  |  | |  | |  |
| 15. Audit problem | 0.03 | 0.02 | 0.03 | 0.09** | 0.09** | 0.04 | 0.06** | 0.14** | 0.09** | 0.22** | 0.05* | 0.05* | 0.06** | 0.52** |  |  |  | |  | |  |
| 16. Espad BD | 0.04 | 0.02 | 0.03 | 0.09** | 0.11** | 0.02 | 0.09** | 0.22** | 0.15** | 0.30** | 0.04 | 0.05 | 0.04 | 0.75** | 0.42** |  |  | |  | |  |
| 17. Age onset SA | 0.01 | 0.01 | 0.02 | 0.10** | 0.10** | 0.1** | 0.08** | 0.15** | 0.23** | 0.24** | 0.09** | 0.10** | 0.09** | 0.32** | 0.22** |  | 0.32** | |  | |  |
| 18. Marijuana SA | -0.01 | 0.00 | 0.01 | 0.00 | 0.01 | 0.01 | 0.02 | 0.26** | 0.23** | 0.35** | 0.12** | 0.13** | 0.11** | 0.39** | 0.22** |  | 0.38** | | 0.62** | |  |
| Mean | 0.53 | 0.12 | 0.28 | 0.89 | 3.70 | 0.70 | 0.60 | 0.91 | 0.55 | 0.47 | 4.46 | 0.86 | 0.51 | 1.09 | 0.36 |  | 0.51 | | 0.51 | | 0.52 |
| SD | 1.53 | 0.87 | 0.56 | 1.23 | 5.34 | 1.91 | 0.66 | 1.78 | 1.90 | 1.04 | 6.79 | 2.73 | 0.90 | 1.51 | 1.21 |  | 1.82 | | 1.82 | | 2.19 |

Note: *p<.05; **p<.01

Table B. Correlations between covariates (personality and neural) with compulsive and externalizing behaviors (N=1938).

|  | Neuroticism | Extraversion | Conscientiousness | Novelty seeking | VBM  Right OFC | VBM  Right VS | VBM  Left OFC | VBM  Right DLPFC | VBM  Left BA10 | VBM  Left BA9 | VBM  Left MFG |
| --- | --- | --- | --- | --- | --- | --- | --- | --- | --- | --- | --- |
| 1. OCD symptoms | 0.27^**^ | -0.11^**^ | -0.01 | -0.04 | -0.01 | -0.02 | -0.01 | 0.00 | 0.00 | -0.01 | 0.00 |
| 2. OCD impact | 0.06^*^ | -0.03 | 0.04 | -0.04 | -0.02 | -0.02 | -0.01 | -0.01 | 0.00 | -0.01 | -0.01 |
| 3. OCD band | 0.28^**^ | -0.12^**^ | -0.04 | -0.06^*^ | -0.01 | -0.01 | 0.00 | 0.00 | 0.01 | 0.00 | 0.00 |
| 4. ED general | 0.35^**^ | -0.03 | -0.02 | 0.05 | -0.09^**^ | -0.11^**^ | -0.09^**^ | -0.10^**^ | -0.08^**^ | -0.10^**^ | -0.11^**^ |
| 5. ED symptoms | 0.31^**^ | -0.01 | 0.00 | 0.06^**^ | -0.11^**^ | -0.13^**^ | -0.11^**^ | -0.12^**^ | -0.10^**^ | -0.13^**^ | -0.13^**^ |
| 6. ED impact | 0.19^**^ | 0.01 | 0.02 | 0.04 | -0.07^**^ | -0.09^**^ | -0.07^**^ | -0.08^**^ | -0.07^**^ | -0.08^**^ | -0.08^**^ |
| 7. ED band | 0.33^**^ | -0.02 | 0.01 | 0.05^*^ | -0.10^**^ | -0.12^**^ | -0.10^**^ | -0.11^**^ | -0.09^**^ | -0.11^**^ | -0.12^**^ |
| 8. CD symptoms | 0.06^*^ | 0.02 | -0.17^**^ | 0.18^**^ | -0.06^*^ | -0.06^**^ | -0.07^**^ | -0.07^**^ | -0.07^**^ | -0.07^**^ | -0.07^**^ |
| 9. CD impact | 0.07^**^ | -0.03 | -0.17^**^ | 0.12^**^ | -0.05^*^ | -0.04 | -0.06^*^ | -0.05^*^ | -0.06^*^ | -0.05^*^ | -0.05^*^ |
| 10. CD band | 0.05^*^ | 0.03 | -0.21^**^ | 0.23^**^ | -0.04 | -0.04 | -0.05^*^ | -0.05^*^ | -0.05^*^ | -0.05^*^ | -0.05^*^ |
| 11. ADHD symptoms | 0.08^**^ | 0.00 | -0.27^**^ | 0.17^**^ | 0.03 | 0.03 | 0.03 | 0.03 | 0.02 | 0.04 | 0.03 |
| 12. ADHD impact | 0.05^*^ | -0.05 | -0.19^**^ | 0.10^**^ | 0.00 | 0.01 | -0.01 | 0.01 | -0.01 | 0.01 | 0.01 |
| 13. ADHD band | 0.08^**^ | -0.010 | -0.26^**^ | 0.15^**^ | 0.03 | 0.04 | 0.03 | 0.03 | 0.02 | 0.04 | 0.03 |
| 14. Audit frequency | 0.08^**^ | 0.09^**^ | -0.20^**^ | 0.31^**^ | -0.05^*^ | -0.05^*^ | -0.05 | -0.07^**^ | -0.05^*^ | -0.06^*^ | -0.07^**^ |
| 15. Audit problem | 0.10^**^ | 0.04 | -0.13^**^ | 0.18^**^ | -0.01 | -0.01 | 0.00 | -0.02 | -0.01 | -0.01 | -0.02 |
| 16. Espad BD | 0.08^**^ | 0.08^**^ | -0.18^**^ | 0.31^**^ | -0.07^**^ | -0.07^**^ | -0.07^**^ | -0.09^**^ | -0.08^**^ | -0.08^**^ | -0.09^**^ |
| 17. Age onset SA | 0.06^*^ | 0.03 | -0.13^**^ | 0.16^**^ | 0.00 | 0.00 | 0.00 | -0.02 | -0.01 | 0.00 | -0.01 |
| 18. Marijuana SA | -0.01 | 0.02 | -0.14^**^ | 0.17^**^ | 0.01 | 0.02 | 0.01 | 0.00 | 0.01 | 0.01 | 0.00 |

Note: *p<.05; **p<.01

Table C. Frequencies and distribution of compulsive and externalizing symptoms, as well as personality correlates (N=1938).

|  | Frequencies of reporting any symptoms  or occurrence (%) | Mean | Standard Deviation | Skewness* |
| --- | --- | --- | --- | --- |
| 1. OCD symptoms | 15.10 | 0.53 | 1.56 | 3.88 |
| 2. ED symptoms | 43.10 | 3.70 | 5.34 | 1.44 |
| 3. CD symptoms | 36.60 | 0.91 | 1.78 | 2.98 |
| 4. ADHD symptoms | 60.20 | 4.47 | 6.79 | 2.23 |
| 5. Number of lifetime  occasions having  five or more drinks  in a row. | 26.30 | 0.59 | 1.18 | 2.16 |
| 6. Number of lifetime  occasions of using  marijuana or  hashish. | 6.80 | 0.15 | 0.71 | 6.01 |
| 7. Neuroticism mean | N/A | 1.92 | 0.62 | 0.19 |
| 8. Conscientiousness mean | N/A | 2.30 | 0.56 | -0.03 |
| 9. Extraversion mean | N/A | 2.49 | 0.47 | -0.32 |
| 10. Novelty seeking | N/A | 111.25 | 12.95 | 0.22 |
|  |  |  |  |  |

*: All analyses were carried out using Maximum Likelihood with Robust standard errors (MLR) estimation, which does not depend on assumptions of normality and thus has been shown to perform well when modelling low prevalent behaviors or non-normal data.

N/A : Not applicable.
